# Reversal of liver fibrosis after splenectomy in a patient with advanced schistosomiasis japonica: A case report with 4-year follow-up

**DOI:** 10.1371/journal.pntd.0007174

**Published:** 2019-04-11

**Authors:** Langui Song, Beibei Zhang, Jiahua Liu, Min Wang, Xiaohe Ma, Lifu Wang, Xiaoying Wu, Zhongdao Wu, Tianping Wang

**Affiliations:** 1 Department of Parasitology, Zhongshan School of Medicine, Sun Yat-sen University, Guangzhou, Guangdong, China; 2 Key Laboratory of Tropical Disease Control, Ministry of Education, Guangzhou, Guangdong, China; 3 Provincial Engineering Technology Research Center for Biological Vector Control, Guangzhou, Guangdong, China; 4 Anhui Provincial Institute of Parasitic Diseases, Hefei, Anhui, China; 5 School of Public Health, Fudan University, Shanghai, China; National Institute of Parasitic Diseases, CHINA

## Introduction

Schistosomiasis is a serious parasitic disease caused by blood flukes of the genus *Schistosoma* [1,2]. As one of the 17 neglected tropical diseases listed by the World Health Organization, it presents the greatest public health and global burden, leading to 200 million infections and threatening 800 million in 78 countries worldwide [1,3,4]. China is one of them, but it has been able to decrease morbidity and prevalence of schistosomiasis japonica prominently [5,6]. Estimates show that although the number of schistosomiasis cases in China drops sharply, the number of advanced cases of schistosomiasis japonica sustained and even rose, from 22,786 in 2000 to 30,573 in 2016 [6, 7]. Advanced schistosomiasis is the most severe form of schistosomiasis japonica. This debilitating condition is associated with liver fibrosis and/or cirrhosis, splenomegaly, portal hypertension, ascites, and gastroesophageal varices, leading to disability or even death [2,5,8]. Therefore, advanced schistosomiasis japonica becomes a severe health burden in China. Therefore, the critical issue confronting China in schistosomiasis control is the challenge of advanced schistosomiasis, especially on the road to elimination of this diseases.

## Case report

**Fig 1:**
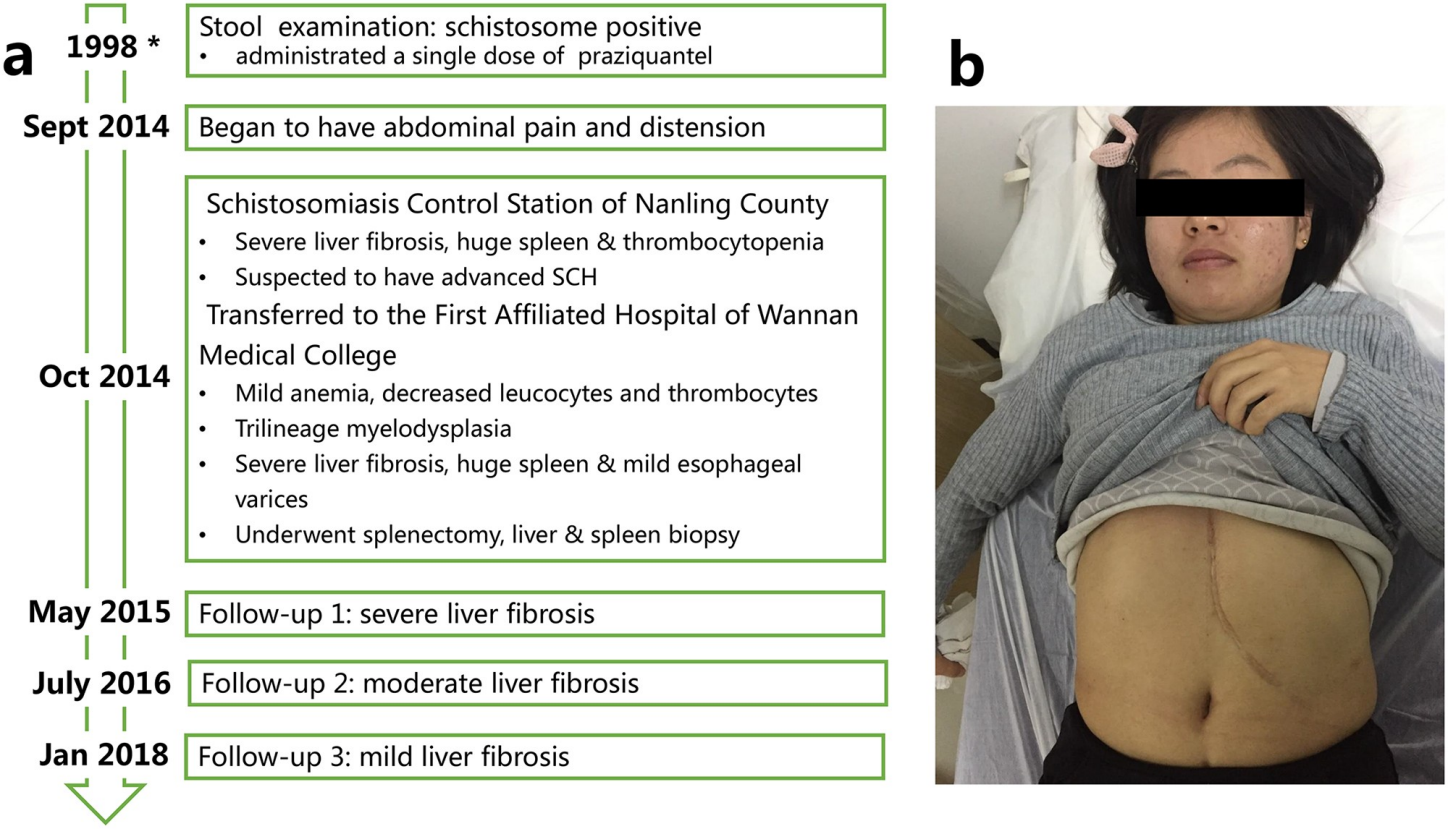
Diagnosis, treatment, and follow-up process of a female patient with advanced schistosomiasis in Nanling (Anhui, China). (a) Diagnosis, treatment, and follow-up process. (b) The surgical incision of splenectomy. *based on the patient’s memory, no medical record; SCH: schistosomiasis.

A 26-year-old female (born 10 September 1988), who lived in Longhu Village, Jishan Town, Nanling County, Wuhu City, Anhui Province of China, an endemic county of schistosomiasis, was complaining of abdominal pain and distension for over 1 month, thus going to the local schistosomiasis-specialized hospital for diagnosis and treatment on 13 Oct 2014. Her diagnosis, treatment, and follow-up process is shown in Fig 1. An abdominal ultrasonography was performed, indicating a mildly enlarged liver with severe fibrosis and a huge spleen. And blood routine showed thrombocytopenia. Due to her symptoms (abdominal pain and distension) and blood test results (a reduction in the platelet count) as well as contact history (living in the endemic area of schistosomiasis), she was suspected to have advanced schistosomiasis and might be in need of surgery as a result of splenomegaly, and thus was referred to the First Affiliated Hospital of Wannan Medical College (Anhui, China) for further diagnosis and treatment. She was admitted to the hospital on 21 Oct 2014 because of liver fibrosis and hypersplenism. Blood routine test showed mild anemia (HB 104 g/L) with decreased leucocytes (white blood cell [WBC] 1.7 × 10^9^/L) and thrombocytes (platelet [PLT] 30 × 10^9^/L). Bone marrow aspiration and biopsy revealed trilineage myelodysplasia. Ultrasounography demonstrated an enlarged liver with severe fibrosis (characteristic fish-scale pattern caused by schistosomiasis), the size of the left lobe of which was 82 × 71 mm and the size of right lobe was 117 mm with an 18-mm–width portal vein (>13 mm considered abnormal, suggesting portal hypertension). Esophagography indicated mild esophageal varices probably resulting from portal hypertension. The length of spleen was 165 mm (>120 mm considered abnormal) while the size was 78 mm (>40 mm considered abnormal).

Hepatitis virus tests were negative (HBV and HCV), and she was a nondrinker and denied long-term use of medications. Despite the fact that no egg was observed in the fecal specimen through microscopic examination, anti-SEA (soluble egg antigen) antibody was positive, which helped to make a clinical diagnosis of advanced schistosomiasis. With the purpose of alleviating portal hypertension and hypersplenism, a splenectomy was performed as well as a liver biopsy on 27 Oct 2014. The section of spleen showed chronic congestive splenomegaly while the section of liver revealed disorganized lobular structure and proliferation of fibrous tissue together with calcified eggs deposition, which provided pathological evidence for diagnosis of advanced schistosomiasis. After operation, blood routine test was performed every 1 to 3 days. A high WBC count (23.8 × 10^9^/L) was observed on 28 Oct 2014, but it decreased steadily and become normal (9.5 × 10^9^/L) on 6 Nov 2014. Whereas, PLT count increased apparently, from 82 × 10^9^/L on 28 Oct 2014 to 887 × 10^9^/L on 6 Nov 2014. Therefore, a low dose of aspirin (0.1 g per day) was prescribed to prevent blood clots at discharge on 6 Nov 2014, and regular blood routine test was recommended to monitor platelet count.

**Fig 2:**
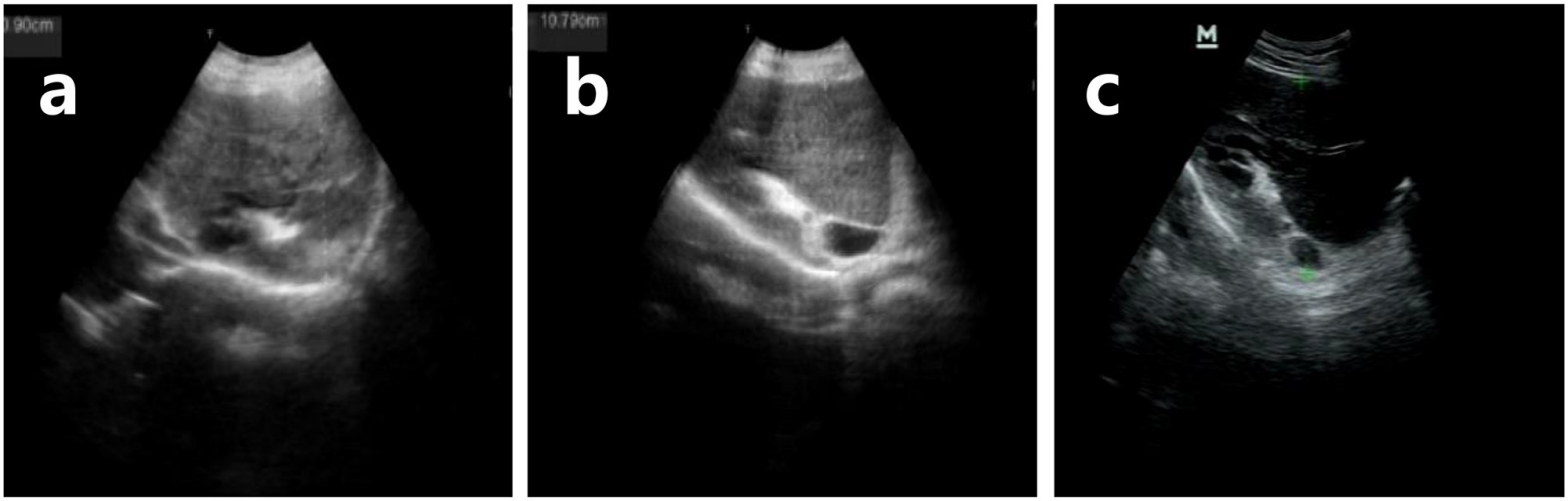
Reversal of liver fibrosis from Grade 3 to 1 within 3 years in a female patient with advanced schistosomiasis after splenectomy in Nanling (Anhui, China). (a) Grade 3, severe fibrosis (checked on 14 May 2015). (b) Grade 2, moderate fibrosis (6 July 2016). (c) Grade 1, mild fibrosis (9 January 2018). The ultrasound examination was performed by the same technician using Teknova for image a and b and Mindray for image c in the Schistosomiasis-specialized Hospital of Nanling (Anhui, China).

She was annually followed up by the local schistosomiasis-specialized hospital because she was entitled to the medical insurance supported by government finance as an advanced schistosomiasis patient. On her first follow-up in 2015, platelet count (362 × 10^9^/L) was nearly normal, and a high gamma-glutamyl transferase (GGT) level (91 U/L, normal range 7–50 U/L) was detected as well as severe liver fibrosis seen from the ultrasonography, and thus glutathione and diammonium glycyrrhizinate were prescribed to protect the liver. On her second follow-up in 2016, both blood and liver routine tests were normal, and the liver fibrosis condition improved to moderate, and therefore she continued to be prescribed liver protection drugs such as glutathione. The patient was suggested to have an abdominal ultrasound examination once a year. Ultrasonography of the liver revealed severe to mild fibrosis (Grade 3 to 1) from the admission examination in 2014 to the third follow-up examination in 2018 (Fig 2, Table 1), indicating the positive effect of the surgery that facilitates the flow of blood so it is less likely to pool in the portal system. The liver is mildly enlarged in size, and its left lobe is shrunk with length dropping from 82 mm to 70 mm and thickness from 71 to 52 mm, though it is still a little oversized, whereas only a small decline was observed in the size of the right lobe (from 117mm to 109 mm).

**Table 1:** Ultrasound results of liver and spleen of the female with advanced schistosomiasis from 2014 to 2018.

Order	Examination date	Liver				Spleen	
		Fibrosis grade	Portal vein width (mm)	Left lobe* (mm)	Right lobe diameter (mm)	Length (mm)	Thickness (mm)
Admission	21 Oct 2014	Severe	18	82 × 71	117	165	78
Follow-up 1	14 May 2015	Severe	10	72 × 59	109	/	/
Follow-up 2	6 Jul 2016	Moderate	10	77 × 52	109	/	/
Follow-up 3	9 Jan 2018	Mild	9	70 × 52	109	/	/

*Left lobe: length × thickness

## Discussion

Advanced stage is the most severe form of schistosomiasis, which accounts for 5% to 10% of all schistosomiasis morbidities [5,8], but its precise developmental mechanism remains unclarified, probably related to long-term exposure to infested water, incomplete treatment, and genetic susceptibility [5]. Schistosome eggs deposition in the liver tissue elicit a granulomatous response, resulting in continuous fibrosis of the periportal tissue, which may progress to induce obstruction of the portal vessels, leading to development of advanced schistosomiasis [8]. Surgical splenectomy has been applied to treat splenomegaly and hypersplenism since the 1950s [9] and is often performed in advanced schistosomiasis cases with megalosplenia and hypersplenism, to attenuate thrombocytopenia [5]. After splenectomy, amelioration of liver fibrosis are observed in murine model in different settings [10,11]. In addition to promoting blood flow to the liver, splenectomy can alter distribution and turnover of many immune cells, such as macrophages as well as platelets [10,12], and it may modulate the immune microenvironment and help the liver cells regenerate [13], which possibly play significant roles in the resolution of liver fibrosis.

Due to the large-scale screening and chemotherapy of schistosomiasis program in China (from the mid-1980s to the early 2000s), all of the primary and middle school students were required to provide a stool sample for schistosome detection [5]. As the patient recalled, she was tested for *Schistosoma japonicum* infection when she was a Grade 5 primary student (11-year-old), was found out to be stool positive, and thus was treated with praziquantel. But the investigators did not follow-up her status. Therefore, she may be not fully cured because the single dose praziquantel may not be able to fully eliminate the worms in her body [14], and/or the egg-granuloma leading to progression of tissue fibrosis, but she may be fully cured, and it could be due to another schistosome infection. After surgery, her condition gradually improved, which is evident by the results of liver ultrasounography from severe fibrosis to mild-to-moderate fibrosis as well as the portal vein from 18 mm to 9 mm. Chronic and advanced schistosomiasis patients are recommended to take antifibrotic drugs regularly, which can help mitigate or even reverse the fibrosis condition. Although long-term antifibrotic medications were prescribed to her, she only administered the given drugs several times, so it’s unlikely to be the major contribution to the relief of liver fibrosis. It seems that splenectomy plays an important role in liver fibrosis improvement by greatly alleviating portal hypertension and promoting blood flow, because the liver has strong and robust regenerative capacity when it is with sufficient blood (oxygen and nutrition) supply [13]. To conclude, early operation and effective decompression of the portal system are probably the major factors resulting in reversal of liver fibrosis for this advanced schistosomiasis case with enlarged spleen, but it only provides some hints for us because we are just learning from a single case, and further studies on more patients receiving splenectomy are needed. Also, the positive change of this case might be partially attributed to her relatively young age and her healthy lifestyle habits that refer to drinking around seven cups of water daily, eating lightly, keeping early hours, and exercising regularly without consumption of alcohol, tobacco, and unnecessary drugs [15,16]. All the above behaviors are beneficial to the liver, avoiding extra burden to this organ.

In the process of schistosomiasis elimination in China, public awareness about the prevention and control of this disease should be consolidated because there are still a large number of chronic infection cases or asymptomatic carriers [6]. They have acquired this infection before without complete deworming of parasites, and despite no noticeable symptoms for now, they might develop advanced schistosomiasis gradually within years due to its insidious onset [5]. This late-stage of the disease is debilitating and potentially fatal, usually requiring long-term medical care and assistance, which is time-consuming and money-taking. The best way to reduce the health cost is to prevent it from occurring. Therefore, regular ultrasound examination is strongly recommended for those in endemic regions, which is noninvasive, convenient, and economic, and it can be acted as a screening tool. Especially for those aged between 20 and 50 years, namely, the workforce; it would be a huge loss to the society if they become the advanced patients. Therefore, public awareness about the prevention and control of this disease should be consolidated in the areas, particularly in the process of schistosomiasis elimination.

## Ethics statement

This study was approved by the Medical Ethics Committee of Fudan University in Shanghai, China (No. IRB# 2016-TYSQ-03-17), and all the procedures were performed in strict accordance with principles expressed in the Declaration of Helsinki. Written informed consent was obtained from the patient for participation of this study and publication of this paper and any accompanying images.

**Box. Key learning points**

1. Schistosomiasis japonica remains one of the most important public health issues in China, and the late-stage is the leading disability stage.
2. Combined with contact history and the results of blood routine test, bone marrow examination, abdominal ultrasound examination, as well as spleen and liver biopsy, the female patient was confirmed with advanced schistosomiasis.
3. Early operation and effective decompression of the portal system may be the major factors resulting in reversal of liver fibrosis for this advanced schistosomiasis case with enlarged spleen.
4. The insidious onset of advanced schistosomiasis may lead to serious outcomes, so that all residents in the endemic regions are advocated to have regular ultrasound examination, which helps improve health at individual and population levels.
